# On the Evil Effects of Marriages of Consanguinity

**Published:** 1857-02

**Authors:** S. M. Bemiss


### SELECTIONS.

### *On the Evil Effects of Marriages of Consanguinity.* A Paper read before the Louisville Medical Club, by S. M. Bemiss, M. D.

* * * "They breed in-and-in, as might be known;
Marrying their cousins, nay, their aunts and nieces,
Which always spoils the breed if it increases."

Some facts illustrating, in a remarkable degree, the evil results of marriages of consanguinity, fell under the observation of the writer several months ago, and determined him to collect others of a similar character, and endeavor to arrange them in such a form as to warrant some definite conclusions upon this important subject. The collection of original facts connected with these marriages is a matter of difficulty and delicacy, and oftentimes as disagreeable as it is difficult and delicate. We can seldom gain information in reference to their history and results from any source so exact and reliable as from the family itself; and such is frequently the secrecy observed by relatives in regard to these revelations, that we are unable to obtain statistics sufficiently minute to afford a basis for positive conclusions. Such statistical information as my paper contains, may, however, be relied upon as strictly correct; for I have at once rejected all presented to me in such a manner that I was not able, from my knowlege of the informant, to indorse as entirely to be relied upon. One other difficulty met with in a comprehensive presentation of this subject, is the poverty of its literature; for, though long and able treatises have been written upon the best methods of preventing or arresting deterioration of domestic animals, by the introduction, from time to time, of new crosses into their respective breeds, yet the question of degradation of numerous families of the human species, by neglect of similar counteracting influences, has rarely been a subject of literary or medical discussion. Nevertheless, the opinion is strictly tenable, however humiliating it may be to our pride, that the rules which govern the procreation of species are not essentially different in man and domestic animals. This want of medical research upon the subject in question, does not proceed from the novelty or recent origin of the truth just stated, for Rilliet has well observed, "No one can claim priority of the idea, when its antiquity is such that we cannot trace it to its source."

In primitive ages, marriages between near blood relations were occasionally necessary to the perpetuation of the race; but it is very probable that their abuse led to the early enactment of laws to establish the degrees of consanguinity, beyond which marriages might be consummated without fear of perverted sanitary condition in the progeny. It is by no means an ascertained fact of history, that these early laws of incest owed their origin to physiological observations; but it seems to me entirely reasonable to infer that, witnessing the pernicious effects of such unions upon offspring, first suggested the establishment of laws to prevent the continued repetition of the evil. Those were ages in which physical force had not been superceded by the inventions and mechanical appliances of the present day; and the power of a community was measured by the amount of muscle under its control. Legislators, who contended that "walls of men were better than walls of stone," would certainly exercise the most sedulous care to maintain, so far as practicable, the physical integrity of their people. In further proof of this inference, Socrates, when inveighing against the incestuous marriages that were sometimes practised at Sparta and Athens, says, he regards them as "prejudicial to the healthy propagation of the species."—(Rilliet.)

The laws of Moses interdicted marriages within the third degree of relationship. The Roman laws were more strict in this respect in former than in later times. Plutarch says, "In ancient times the Romans abstained from marrying their kinswomen in any degree of blood; as they at present forbear their aunts and sisters. It was late before the marriage of cousins-german was dispensed with."—(Janeway.) The Catholic Church, at an early period, opposed itself to blood alliances. Pope Gregory, the Great, in proscribing such marriages, gives, like Socrates, a physiological reason: "Experimento didicimus ex tali conjugio sobolem saccrenere non posse."—(Rilliet.)

References may be found to the unfortunate influences of marriages of consanguinity upon offspring, in various medical works of the previous and present century,* but no facts are adduced to support the conclusions of the authors; nor have any statistics, illustrating these effects, been presented to the profession, so far as I am aware, except some facts in Dr. Howe's valuable reports on idiocy.

By much labor, I have obtained statistical accounts of thirty-four marriages of consanguinity. Of this number, twenty-eight were of the third degree of the civil law, or between first cousins. Of the total number of marriages, twenty-seven were fruitful and seven sterile. The twenty-seven fruitful unions, produced one hundred and ninety-one children. In only thirteen of these marriages, was the sex of the offspring reported, giving forty-nine males to forty-two females. Of the twenty-eight marriages of the third degree of relationship, twenty-three were fruitful and five sterile. Of the six marriages in the fourth degree, four were fruitful and two sterile. Of the six of these latter instances of sterility, the female was the product of a marriage of consanguinity. The relative proportion of children to the total number of marriages was as 1 to 5-6. The average fecundity to each fruitful union was seven and a slight excess. The average fecundity to each fruitful union was seven and a slight excess. The average births to each fecund marriage in the third degree of kinship was 6-87, nearly. The average number of births in the fruitful unions of the fourth degree, was 8½.

Of the 102 children resulting from these marriages, 58 perished in early life. In 24 of the 58 deaths, the causes are stated as follows: of consumption, 15; of spasmodic affections, 8; of hydrocephalus, 1. Of the 134 who arrived at maturity, 46 are reported as healthy; 32 are set down as deteriorated, but without absolute indications of disease; and 9 are returned without any statement as to health or condition. The remaining 57 all possess such abnormities as to render them the subjects of

* Mason Good, Combe, Barlow, &c.

particular observation. These are classed as follows: 23 are scrofulous; 4 are epileptics; 2 are insane; 2 are mutes; 4 are idiots; 2 are blind; 2 are deformed; 5 are albinoes; 6 have defective vision; and 1 has chorea.

In point of fecundity, these marriages probably present results not differing materially from the average fertility of marriages in the rural districts of the West. I do not know of any rules by which to determine the average number of births to a marriage among our country population, but I do not doubt they will equal in fruitfulness the most prolific portions of Europe. In some villages of Scotland, the proportion of births to a marriage is stated to be seven. (Cy. Pract. Med.) The parties to the above intermarriages were, with one or two exceptions, of rural habitation; were, in many instances, remarkable for mental and physical development; and were all surrounded, in a large majority of cases, by circumstances of living calculated to insure the utmost degree of fruitfulness and prolonged life.

These statistics, then, I am satisfied, exhibit results on this account more than usually favorable to the offspring. In support of this opinion, we may contrast this report with Dr. Howe's observation of 17 marriages of blood relations, in his report on idiocy. These 17 marriages gave "birth to 95 children, of whom 44 were idiots, 12 scrofulous and puny, 1 deaf, 1 dwarf, 58 in all, of low health or imperfect, and only 37 of even tolerable health." An unusually large number, over one-fifth, of the marriages in my report, were sterile; and I am not aware that this can, in any instance, be imputed to other causes than the influence of consanguinity. Some of the parties to these sterile unions have had excellent corporeal and mental endowments, and have arrived at unusual longevity. In four instances reported to me, females descended from these intermarriages have proved barren without exhibition of constitutional defect. In two of these instances, they had married relatives; in the other two, they married without the circle of family affinity.

I shall not attempt to offer any hypothesis as to the active cause of sterility in these cases. It is a subject in reference to which physiological reasoning has, up to the present time, furnished no satisfactory results. We cannot force our researches into the hidden penetralia of nature, and there discover how her processes of reproduction are so interfered with, as to render these intermarriages disastrous to their issue; nor by what means she avoids these unfortunate results, by rendering many such unions fruitless. We must leave the development of this subject to the future physiologist: if, indeed, the sea of human knowledge shall ever extend its limits in this direction.

I come now to speak of the defects of the offspring, in the cases alluded to. I have no doubt that the number of cases of scrofula is larger than the same number of descendants of marriages of consanguinity would ordinarily exhibit. This predominance of scrofula probably results from the fact that, in three of the families, there is reason to suspect the previous existence of strumous taint. These families alone yield sixteen cases of scrofula. This fact, and the large proportion of the deaths ascribed to phthisis, demonstrate the truth of Dr. Barlow's observation, that the tuberculous diathesis "shows itself, in the greatest intensity, in the offspring of marriage between relations, in whose family the taint has already existed."

I have not obtained sufficient information to enable me to describe the particular manifestations of scrofulous diathesis in each individual case; some of them are, however, represented as scrofulous ophthalmia.

Rilliet places epilepsy first in order of frequency, of the diseases of the nervous system, to which the products of family intermarriage are most liable. My report comprises four epileptics; the father of one of whom was likewise the result of a marriage of cousins. Eight of the deaths are ascribed to spasmodic affections, and four of this number are specified as epilepsy.

I have no information in regard to the degree or character of mental aberration of the two insane subjects, contained in the enumeration. The two cases of muteism were congenital, and will be again referred to, as will also the four cases of idiocy.

I am not informed whether the two cases of blindness were congenital, or produced causes occurring after birth; they, however, both occurred in the same family, and were, in all probability, congenital.

The cases of deformity were, in one instance, curvature of the spine; in the other, malformation of an arm. The six cases of defective vision, were myopia, and the results of scrofulous ophthalmia.

It now remains to notice the most remarkable of all the abnormities of offspring my report presents. I refer to albinoism. Absence of the pigmentary cells of the integument and coats of the eye, is, undoubtedly, like most other deviations from the normal type of reproduction, a mark of deterioration. But albinoism sometimes occurs where there is no cause or condition, apart from its own manifestation, to lead us to infer degradation; and, having once occurred in a family, the influence of maternal emotion might have much to do with its repetition. It may exist in connection with a moderate state of corporeal health, and a striking development of intellectual sprightliness, as in some of the subjects of my report. In domestic animals, it is so well understood to indicate impaired value, that connoisseurs in horseflesh reject such animals as have absence of coloring pigment in the hair and tegumentary tissues. M. Siéele's* observations upon albino cats led him to conclude that it was, in this animal, uniformly attended by absence of the auditory sense. No defect of hearing has accompanied its presence in the subjects of my report. Almost all the albinos here included are nearsighted. Esquirol also mentions an albino who was certainly myopic, and another whom he supposed to be. I am not aware that any author has spoken of this condition as the result of in-and-in marrying; but Esquirol states, that wherever we meet with albinos, there we also find the goitrous and idiotic; and these latter are known to be common products of marriages between kindred. From Dr. Howe's investigations on this subject, it is safe to infer that idiocy is oftener the result of this cause, than was heretofore suspected. In Dr. Howe's report, seventeen out of three hundred and fifty-nine idiots, were found to be products of marriages of consanguinity. If the same ratio held good throughout this country, there would have been, in 1850, seven hundred and forty-seven idiots in the United States, the offspring of parents connected by blood-ties.

Congenital deafness is another impairment common to the products of marriages of this character. M. Meniese, physician to the Imperial In-

---

* London Medical Times, vol. 18, p. 128.

stitute for the Deaf and Dumb, at Paris, considers this infirmity, when congenital, more often referable to this cause than any other. My inquiries have not, thus far, led me to a similar conclusion; but its great frequency among the offspring of such marriages, cannot be doubted. A writer in the National Intelligencer, some years ago, stated, that "a great proportion of the inmates of the asylums for the deaf and dumb, the blind and idiotic, are found to be the product of the intermarriages of cousins." Then, if we add to the 747 idiots, the mute, epileptic, blind, and insane products of these marriages, computing the number at the very lowest probable figure, and sum together the whole, we shall have, resulting from the physiological error in the United States, not less than *two thousand* victims, whose conditions are, for the most part, irremediable; besides a greater number suffering from other impairments of constitution entailed upon them by the same cause. Truly,

"Democritus did well to laugh of old:
Good cause he had, but now much more;
This life of ours is more ridiculous
Than that of his, or long before."

I am aware that many circumstances may produce defective issue besides the influence of consanguinity, such as the habits of the parents, and their particular condition at the period of approach. Maternal emotion or constitutional derangement during pregnancy, may also frequently determine the character of the offspring But these accidental influences are not more likely to prevail in marriages of consanguinity, than in those of a different character; while the number of departures from a healthy standard, in the issue of these marriages, is incomparably greater than in those where no such connection exists.

To prove still more certainly that these various impairments of offspring are due to the influence of too frequent admixture of the same blood, we have only to associate the cases in my report in the various degrees of relationship, and observe if the proportion of accidents to the offspring is increased with the degree of relationship. To arrive at this point, I shall divide the productive marriages in my report into three classes: those of the fourth degree, or between second cousins; those of the third degree, or between first cousins; and those nearer than the third degree, as between double cousins, or between cousins, themselves the descendants of cousins. The table will stand thus:

| Degree. | No. of Marriages. | No. of Children. | Died. | Diseased. | Deteriorated. | Healthy. | Unknown. |
|---|---|---|---|---|---|---|---|
| 4th degree, | 4 | 34 | 8 | 6 | 10 | 10 | |
| 3d degree, | 19 | 130 | 37 | 31 | 17 | 36 | 9 |
| 3½ degree, | 4 | 27 | 13 | 5 | 6 | 3 | |

It will at once be seen, that the percentage of calamitous results to the progeny is largely increased as the relationship becomes closer. I have also accounts of two marriages in the second degree, or between uncles and neices, but they have not been consummated sufficiently long to determine their results.

I now propose to notice briefly the circumstances calculated to modify the effects of marriages of consanguinity, either in lessening or aggravating their evil results. That these marriages are sometimes practiced without apparent ill consequences to the offspring, is obvious to all whose attention is drawn to the observation of these points. Under what circumstances, then, may they be expected to be followed by healthy offspring? Is it when, as Mercatus has advised those entering upon matrimony, the parties are of opposite temperaments? I incline to the opinion that this, with some other circumstances to be mentioned, has great influence in determining the results. Baillou has remarked, that "parents transmit to their children disease as well as wealth, but the former much more certainly than the latter." (Stillé's Pathology.) This remark is probably equally true with regard to points of temperament and types of physiological idiosyncrasy as to predisposition to disease may often consist in a hyper-manifestation of certain constitutional peculiarities. Nature seems to have designed that the conditions and tendencies of human organisms should be kept very nearly in a state of equilibrium. This equipoise, necessary to the healthy condition of man, upon whatever inexplicable cause it may depend, may be readily disarranged by giving undue predominance to any particular constitutional phase. The slighter deviations from a normal mean would constitute individual or family peculiarities; while more marked perversions become morbid manifestations, and infirmity results. As in the moral man none are exempt from the taint of sin, so in the physical man each individual of our race has his obliquity towards disease,—generally, perhaps uniformly, towards some particular disease. It is then reasonable to expect that when two individuals marry, who possess the same morbid proclivity, their offspring will present that identical divergence, but in a much more marked degree. Thus, undoubtedly, have originated many family peculiarities, perverted tastes, and morbid diatheses. The circle of individuals thus constituted sometimes includes large communities, as is observed in reference to the disease denominated Plica Polonica, which attacks the Polish and spares the Russian peasant living under external circumstances equally liable to produce the malady. (Pritchard.) There is no better mode of maintaining this happy mean of temperament than by admixture of types of constitution possessing no family identity. We may possibly and justly, think with Benton, that "it hath been ordered by God's especial providence, that in all ages there should be (as usually there is,) once in 600 years, a transmigration of nations, to amend and purify their blood, as we alter seed upon our land; and that there should be, as it were, an inundation of those northern Goths and Vandals, which overran, as a deluge, most parts of Europe, to alter, for our good, our complexions, which were much defaced with hereditary infirmities, which, by our lust and intemperance, we had contracted."

History presents some instances altogether antagonistic to the doctrine of degradation from this cause. Especially does Sacred Writ offer some cases, of such remarkable emphasis, that it would be extremely difficult to explain the non-sequence of deterioration of offspring, if the rule by which our contemporaries are measured were equally applicable to them. Abraham, Isaac, and Jacob, all married wives connected to them by blood-ties, and yet they were the chosen progenitors of a privileged and highly gifted people, and nothing pertaining to their history suggests the idea of immediate degeneration of progeny. A very learned divine has explained to me this seeming contravention of a natural law, by the supposition that, as the Jews were a people chosen for an especial purpose, they existed under abnormal conditions, and all influences ordinarily enuring to their prejudice were providentially countervailed. This presumption is certainly plausible; and as we see the same people protected in future trials by miraculous interposition, the argument may be entertained without violence to reason. But without presupposing the exercise of supernatural influences, the apparent inoperativeness of this law of degradation by in-and-in marrying, in the above instances, as well as in those of Adam's sons and daughters, may be explained by the incomparably superior endowments of these primeval denizens of our globe. Those were days in which man dwelt as it were in the presence of his Creator; "when," as Schlégel observes, "God familiarly taught man the rudiments of speech, as a parent teaches a child." If those ancient patriarchs were found worthy of these high honors, and if they could ordinarily attain to a longevity many times beyond that to which our most perfect organizations can now arrive, how immeasurably superior to ourselves must we suppose them to have been in vigor and excellence of constitution. They were thus enabled to propagate the race by alliance with their own blood, with no such baneful results as similar causes now call forth. But it is not necessary to believe that such unions were even then exempt from pernicious effects, but in their perfect condition of corporeal organism, and freedom from predispositions to disease, the deterioration of race resulting from this cause was probably exhibited in the lowering of the vital powers and simple abreviation of life, rather than in demonstrations of the diseases of the present day. We still see these very same circumstances of constitution and condition counteracting, to a limited extent, the evil tendency of this cause. In the early settlement of the West, the inhabitants were, for security, gathered into communities of greater or less magnitude, separated from each other and from the older States by miles of dangerous wilderness. It was natural that each community should be composed, in a great degree, of blood relations, since, in forming companies for migration, the several branches of one family would often. When in their new homes, a scarcity of marriageable material would often render unions between relations expedient, and afterwards, these covenants, arising at first from necessity, became a habit, often convenient in some respects, since it preserved estates within the family circle. But these pioneers were a hardy, robust people, living much in the open air, and undergoing vigorous exercise; having for their aliment wild game and the fresh products of a genial soil, and not addicted to any habits calculated to impair the integrity of their well-endowed constitutions. We would naturally expect conditions of life so favorable to the sound development of the bodily organism to overrule all counteracting influences, and it might prove almost the exception, when marriages of consanguinity gave origin to defective issue. Not so, however, among the valleys of the Alps, where from the barriers formed by impassable mountains, the same seclusion of communities and frequency of family alliances are found to exist. There, with impure air, bad diet, and constitutions infected for generations with hereditary taints, it proves an exception when a healthy child is born from parents of the same blood. There we find goitre, cretinism, scrofula, albinoism, and muteism in their most aggravated forms.*

In this country, the Jews, whose religion forbids them to marry except with their own race, are often driven, from the scarcity of marriageable subjects in their communities, to form alliances with their own kindred, and a physician of distinction informs me that they are peculiarly liable to strumous taints and congenital defects. In this connection it may also be of interest to mention Bayard Taylor's observation, that the Jews of America are far inferior in personal appearance to the Jews of Palestine. These same social conditions and connubial exigencies exist also among the free colored inhabitants of the Northern States, and may account for the increased ratio of deaf and dumb, blind and insane, found in this population. (Comp. U. S. Census, 1850, p. 77.)

Wherever in-and-in marriages are practised under circumstances of life calculated to annul their tendency to deprave the offspring, we see a prominency given to certain points of physiognomy, which constitute striking marks of resemblance, not only evinced in families, but sometimes found to pervade a numerous nation. Thus Hippocrates states that the Scythians all resembled each other, although they were different in appearance from all other people. The Jewish face has, under favorable circumstances, retained its characteristics from the earliest ages to the present day.

History will, I think, sustain the opinion that the most vigorous people have sprung from the ingrafting of nations differing in constitution and temperament from each other. I believe, with an observing writer, that the extraordinary activity and energy of the American people are due to the composite nature of their blood. This rule, however, seems subject to some qualification; for there certainly exist strong reasons to believe that matrimonial alliances between the greatest possible contrasts to be found on our globe, the negro and Caucasian races, for instance, are not favorable to the most vigorous propagation of species. I do not look upon mulattoes as hybrids, but think they exhibit less of vigor and vital force than are found in crosses where there is less contrast. Mr. Alexander, perhaps one of the most experienced cattle-breeders of the world, has observed that the cross between the finest and coarsest breed of cattle is far inferior to that between the best blood and a medium between that and the worst.

The prevention of the serious evils I have under consideration, which of course consists in the prevention of in-and-in marriages, is a point which I do not think is to be attained by enactments of civil laws bearing upon the subject. It would prove equally difficult to convince either legislators or communities that there could be a necessity for going beyond the requirements of the Levitical law. It is, however, the duty of physicians to labor unremittingly in the collection of facts pertaining to this subject, and to submit them to the public. A reasoning man will refrain from a connubial association likely to inflict irreparable

* It may be proper to remark, in this connection, that there is within a few miles of this city, a child, the issue of cousins, whose cranial bones have undergone the rachitic softening so frequently observed among the cretins and idiots of the Alps.

injury upon his offspring, after he has learned that such is the fact; and church governments will forbid their clergy to celebrate nuptials which they find tend to the abasement of the species and the subversion of God's beneficence to mankind. As previously stated, the Catholic Church has already discouraged union in any near degree of blood affinity. We have no means of ascertaining, by reference to masses of population in diminishing the congenital occurrence of such defective offspring as my report presents. It has been attempted in Europe, as Rilliet mentions, with results favorable to Catholic populations. Quetelet's computation of the ratio of deaf and dumb to the whole population is precisely the same in both England and Italy. M. Meniese states that at this day every trace of this interdiction of the Church has disappeared: "Pendant une longue suite de siècles, le mariage fut absolument interdit á tous les individus parents á un degré quelconque, l'Eglise se réservant le droit d'enfreindre la règle posée par elle méme dans les rare circonstances dont elle voulait apprécier la valuer, mais ces rigueurs de la discipline furent sujette, comme tout d'autres choses, à un rélanchment déplorable, et aujourd'hui toute trace de ces interdictions a disparu."

The facts offered in my paper are much too meagre to afford a basis for positive conclusions, but I think it is a step in the right direction, and will at least serve as a guide for other inquiries on this subject. I have, in the last few days, learned, with much pleasure, that Dr. John Bartlett, of Keokuk, Iowa, is now collecting facts for the publication of an essay on this subject. We may congratulate the profession that such an earnest and indefatigable lover of science has taken the subject in hand. The attention of the public press is somewhat, but not sufficiently, awakened to the importance of this subject. The Ohio Legislature, much to the credit of her physicians and law-givers, passed, at their last session, a law requiring the assessors, throughout the State, to collect the facts connected with marriages of consanguinity and certain defects of offspring, and report them. These statistics will furnish a sufficient amount of testimony to establish, beyond cavil, the character and degree of influence these marriages exert upon offspring, and the profession will await with much anxiety, the publication of this most important addition to our vital statistics.*

For reasons too apparent to be indicated, I am compelled to forego the satisfaction of an individual expression of thanks to many friends, both in and out of the profession, for assistance they have kindly rendered me in the collection of facts upon this subject.

---

* The following is a copy of the Act referred to:

An Act to ascertain the Number and other Facts respecting Deaf and Dumb, Blind, Insane, and Idiotic Persons, in the State of Ohio.

SECTION 1. Be it enacted by the General Assembly of the State of Ohio, That the Assessors in the several townships of each County of the State, while performing their duties, shall ascertain and enter upon a schedule prepared for the purpose, the name, in full, of each deaf and dumb, blind, insane, and idiotic person in the township, together with the age, sex, color, occupation, and place of birth of said persons, and whether educated or not; also, the names, in full, of the parents of said deaf and dumb, blind, insane, and idiotic persons, their place of birth, occupation, number of children, number of deaf and dumb children, and what affinity of blood, if any, existed between the parents previous to marriage; and that said papers be returned, in due form, to the Auditor of the proper County, at the time of returning the assessment of property, and by the said Auditor to the Secretary of State, on or before the first day of July, 1856. The Auditor of State shall furnish to the several County Auditors the necessary blanks or schedules to carry out the provisions of this Act.—*North American Md. & Churg. Review.*

